# Regional brain development analysis through registration using anisotropic similarity, a constrained affine transformation

**DOI:** 10.1371/journal.pone.0214174

**Published:** 2020-02-24

**Authors:** Antoine Legouhy, Olivier Commowick, Maïa Proisy, François Rousseau, Christian Barillot

**Affiliations:** 1 CNRS, INRIA, INSERM, IRISA UMR 6074, Empenn ERL U-1228, Univ Rennes, Rennes, France; 2 Radiology Department, CHU Rennes, Rennes, France; 3 IMT Atlantique, LaTIM U1101 INSERM, UBL, Brest, France; Center for Neuroscience and Regenerative Medicine, UNITED STATES

## Abstract

We propose a novel method to quantify brain growth in 3 arbitrary orthogonal directions of the brain or its sub-regions through linear registration. This is achieved by introducing a 9 degrees of freedom (dof) transformation called anisotropic similarity which is an affine transformation with constrained scaling directions along arbitrarily chosen orthogonal vectors. This gives the opportunity to extract scaling factors describing brain growth along those directions by registering a database of subjects onto a common reference. This information about directional growth brings insights that are not usually available in longitudinal volumetric analysis. The interest of this method is illustrated by studying the anisotropic regional and global brain development of 308 healthy subjects betwen 0 and 19 years old. A gender comparison of those scaling factors is also performed for four age-intervals. We demonstrate through these applications the stability of the method to the chosen reference and its ability to highlight growth differences accros regions and gender.

## Introduction

**Fig 1:**
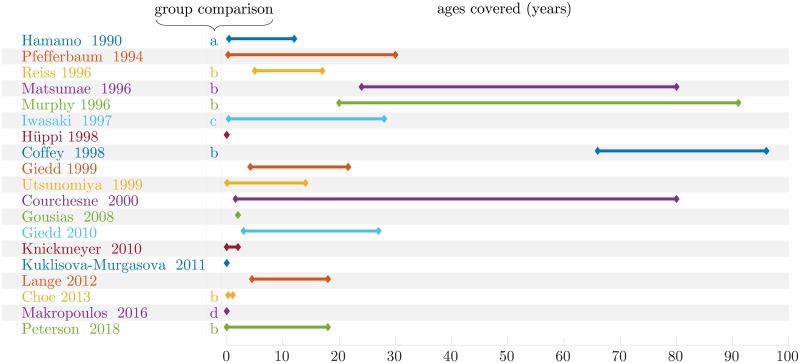
Ages covered by different brain volumetric studies, group comparison between: a—Normal / mental retardation, b—Male / female, c—Normal / motor disturbances, d—Normal / premature. [11, 13–30].

In pediatric image analysis, the study of brain development provides insights in the normal trend of brain evolution and enables early detection of abnormalities. Many types of morphometric measurements based on structural images have been explored and have shown their reliability as biomarkers in clinical use as established in [1–6]. Evaluated on a database of subjects covering a period of interest, it allows to better model the brain development and to highlight changes in growth, shape, structure, etc. Those measurements can be conducted on geometrical objects of different dimensions. Unidimensional ones such as the bicaudate ratio (minimum intercaudate distance divided by brain width along the same line) have been explored in [7] and [8] but also biparietal, bifrontal and transverse cerebellar diameters in [9]. Surface-based quantities such as cortical surface area in [10], corpus callosum mid-sagittal area in [11] or cortical folding metrics as in [12] have also been studied. However, the vast majority of studies are based on 3D features through the assessment of region of interest (ROI) volumes. Volumetric measures of different regions of the brain have been considered for specific ages or various temporal ranges. A far from exhaustive list is presented in Fig 1.

Studied regions are very heterogeneous from large areas such as the whole brain itself, cerebellum, lobes or partitions of those to smaller ones such as basal ganglia, hippocampus, thalamus sometimes even separated according to the composition of their tissues (white matter (WM), gray matter (GM), cerebro-spinal fluid (CSF)). Some group comparisons have also been performed mostly between male and female or between preterm and term newborns.

Morphometric measurements can be determined manually. However, this requires the intervention of a medical expert able to select specific landmarks or perform segmentation. These tasks are highly time consuming with a potentially large inter-expert variability. Advances in computational medical imaging allow nowadays the use of semi-automated (requiring some human intervention) or fully-automated techniques. They involve algorithms able to automatically perform operations such as registration and segmentation.

A major drawback of purely volumetric measurements is that they do not provide any information on the shape of the regions or about the anisotropy of their development. In this paper, a new method is proposed that aims at quantifying global and regional brain growth in three arbitrary orthogonal directions of the brain (or ROI) through linear registration. To do so, a transformation called anisotropic similarity is introduced in section 1. It is an affine transformation with scaling directions constrained by orthogonal vectors arbitrarily chosen. A method to estimate, in a 3 dimensional space, the optimal anisotropic similarity for the least squares problem of distances between two sets of paired points is presented in section 2. Those results will then be used to create a registration algorithm based on this transformation. By registering a database of subjects onto a common basis (i.e. an atlas segmented in different ROIs) using anisotropic similarity, we have the opportunity to extract global or regional scaling ratios for all those subjects along arbitrary chosen orthogonal directions.

A direct application is, using the pipeline exposed in section 3, the exploration of regional scaling ratios growth charts along three fixed orthogonal directions through the ages highlighting anisotropic brain development. Resulting curves for whole brain and ROIs (lobes, basal ganglias, cerebellum…) are presented in section 4.2. A comparison of scaling factors from males and females is performed for four different age-intervals between 0 and 19 years old in section 4.3. Finally, the influence of the common reference image on the resulting scaling factors is studied in section 4.4.

Anisotropic similarity registration algorithm as well as other image processing tools used in this paper are publicly available in Anima (open source software for medical image processing: github.com/Inria-Visages/Anima-Public/ - RRID:SCR_017017).

## 1 Theoretical background

### 1.1 Generalities about linear transformations and anisotropic similarity

An affine transformation is a composition of a linear map *A* (*N* × *N* matrix) and a translation *t* (in $R^{N}$) operating on coordinates: *y* = *Ax* + *t*. Using singular value decomposition (SVD) on *A*, we obtain:

$$\begin{array}{c} A=VDW^{T} \end{array}$$ (1)

where *W* and *V* are unitary matrices and *D* is a positive diagonal matrix. By introducing *R* = *V*.Det(*V*), *U* = *W*.Det(*W*) and *S* = Det(*V*)Det(*W*)*D*, we get a modified decomposition:

$$\begin{array}{c} A=RSU^{T} \end{array}$$ (2)

where *U* is a rotation matrix defining the directions of scaling, *S* is a diagonal scaling matrix and *R* is a rotation matrix. We define a new linear transformation, hereafter named anisotropic similarity, which is an affine transformation with constrained directions of scaling. In other words, we define an anisotropic similarity transformation as an affine one where *U* is fixed. Summing up, we have the following in 3D space:

- An affine transformation has 12 degrees of freedom:
  - a rotation (3 dof): the matrix *U* determines scaling directions.
  - an anisotropic scaling (3 dof): matrix *S*.
  - a rotation (3 dof): matrix *R*.
  - a translation (3 dof): vector *t*.
- For an anisotropic similarity, the directions of scaling defined by *U* are constrained. This leaves 9 dof: 3 for rotation, 3 for scaling and 3 for translation.
- For a similarity, the scaling part is constrained to have identical values on the diagonal i.e. *S* = *s* Id with s∈R leading to a linear part of the form *sRU*^*T*^. This leaves 7 dof: 3 for rotation, 1 for scaling and 3 for translation.
- For a rigid transformation, the scaling part is constrained to identity leading to a linear part of the form *RU*^*T*^ which is a rotation matrix since rotation matrices form a group for matrix multiplication. This leaves 6 dof: 3 for rotation and 3 for translation.

### 1.2 Generalities about linear registration

Registration consists in finding an optimal transformation that matches a moving image onto a reference image. This transformation is usually obtained by maximizing a similarity criterion. Many rigid (or linear in general) registration methods have been developed. They can be divided into two families: the ones that try to match geometrical features such as contours or surfaces, and those called iconic that are based on voxel intensities. Some of them use a global similarity measure between the two images such as mutual information in [31] and [32], while others rely on local similarities. Among this second category of approaches, block matching strategies exposed in [33] and [34] have gained in popularity. In those methods, two steps are iterated:

1. Matching: for a set of blocks established in the reference image, homologous blocks best satisfying a similarity criterion are searched in the moving image.
2. Aggregation into a global transformation: an optimization is performed in order to find the global transformation minimizing a distance between the sets of blocks and is then applied to the moving image. Usually, the weighted sum of squared euclidean distance is chosen for the cost function.

In order to perform an anisotropic similarity registration using the block-matching method, the two steps mentioned above have to be iterated. The first one (matching) is performed the same way it would be for any regular linear transformation. It outputs two sets of paired points: *x* and *y* that are in our case the centers of the homologous blocks. The second step (aggregation onto a global transformation) however is dependent on the type of linear transformation we want to determine leading to an adapted optimization in each case.

This optimization step consists in finding, in the set of eligible transformations, the one that best maps *x* onto *y*. Let *x* = {*x*_1_, …, *x*_*M*_} and *y* = {*y*_1_, …, *y*_*M*_} be two sets of *M* paired points coming from the matching step. For a global transformation with linear part *A* and translational part *t*, the least squares problem associated to the matching of *x* and *y* consists in the minimization of the following criterion *C*:

$$\begin{array}{c} C\left(A,t\right)=\sum_{i}{∥y_{i}-\left(Ax_{i}+t\right)∥}^{2} \end{array}$$ (3)

**Remark**. *For the sake of clarity we presented a version with a non-weighted least squares problem but the reasoning is the same with a weighted one*.

The optimal translation $\hat{t}$ can be directly obtained from the optimal linear part $\hat{A}$ (independently of the type of linear transformation) and the barycenters of the two sets of points as developed in [35]. Let $\bar{x}=\frac{1}{M}\sum_{i}^{M}x_{i}$ and $\bar{y}=\frac{1}{M}\sum_{i}^{M}y_{i}$, we have then:

$$\begin{array}{c} \hat{t}=\bar{y}-\hat{A}\bar{x} \end{array}$$ (4)

Let $x_{i}^{\prime }=x_{i}-\bar{x}$ and $y_{i}^{\prime }=y_{i}-\bar{y}$ be the barycentric coordinates. Since $\hat{t}$ can be directly expressed from $\hat{A}$, the problem can then be simplified as the minimization of:

$$\begin{array}{c} C\left(A\right)=\sum_{i}{∥y_{i}^{\prime }-Ax_{i}^{\prime }∥}^{2} \end{array}$$ (5)

In the case of the linear part being affine, there is no constraint. A closed form solution can therefore be easily found as shown in [35]. For rigid and similarity transformations, constraints lead to more complicated lagrangians but a closed form solution can be found as well using unit quaternions in 3D space as a representation of rotations like in [36] and [35].

## 2 Optimal anisotropic similarity between two sets of paired points

To our knowledge, the optimization procedure in the case of anisotropic similarities has not been considered in the literature. We thus present a method also based on quaternions to find the optimal anisotropic similarity between two sets of paired points. Writing an anisotropic similarity *A* as its decomposition: *A* = *RSU*^*T*^ where *U* is fixed, the optimization over variable *A* therefore becomes an optimization over variables *R* and *S*:

$$\begin{array}{c} C\left(R,S\right)=\sum_{i}{∥y_{i}^{\prime }-RSU^{T}x_{i}^{\prime }∥}^{2} \end{array}$$ (6)

Let ${\tilde{x}}_{i}=U^{T}x_{i}^{\prime }$, $\xi_{i}=S{\tilde{x}}_{i}$.

$$\begin{array}{c} \tilde{C}\left(R,S\right)=\sum_{i}{∥y_{i}^{\prime }-R\xi_{i}∥}^{2} \end{array}$$ (7)

*R* can be expressed using quaternions following [36] and [35] and the problem then becomes (see S1.1):

$$\begin{array}{c} \tilde{C}\left(q,S\right)=\sum_{i}\left|\right|y_{i}^{\prime }*q-q*\xi_{i}{\left|\right|}^{2} \end{array}$$ (8)

Where *q* is a unit quaternion and * is the quaternion multiplication. Let *p* and *q* be quaternions, $\bar{p}$ and $\bar{q}$ their conjugated quaternions respectively. There is a matricial representation of quaternions allowing to express quaternion product as a matrix product. Matricial quaternions *P* and *Q* are defined such that: *Q*_*p*_*q* = *p* * *q* and $P_{p}q=q*\bar{p}\Leftrightarrow P_{p}^{T}q=q*p$.

$$\begin{array}{c} Q_{p}=(\begin{array}{cccc} p_{1} & -p_{2} & -p_{3} & -p_{4}\\ p_{2} & p_{1} & -p_{4} & p_{3}\\ p_{3} & p_{4} & p_{1} & -p_{2}\\ p_{4} & -p_{3} & p_{2} & p_{1} \end{array})\;\text{and}\;P_{p}=(\begin{array}{cccc} p_{1} & p_{2} & p_{3} & p_{4}\\ -p_{2} & p_{1} & -p_{4} & p_{3}\\ -p_{3} & p_{4} & p_{1} & -p_{2}\\ -p_{4} & -p_{3} & p_{2} & p_{1} \end{array}) \end{array}$$ (9)

Using those matricial quaternions on $y_{i}^{\prime }$ and *ξ*_*i*_ taken as pure quaternions, we have $y_{i}^{\prime }*q=Q_{y_{i}^{\prime }}q$ and $-q*\xi_{i}=-P_{\xi_{i}}^{T}q=P_{\xi_{i}}q$. Thus, we obtain the following criterion (see S1.2):

$$\begin{array}{c} \tilde{C}\left(q,S\right)=q^{T}(-\sum_{i}{\left(Q_{y_{i}^{\prime }}+P_{\xi_{i}}\right)}^{2})q \end{array}$$ (10)

For further computation, we denote $B_{i}=-{\left(Q_{y_{i}^{\prime }}+P_{\xi_{i}}\right)}^{2}$ and *B* = ∑_*i*_ *B*_*i*_. A lagrangian with unit constraint *q*^*T*^ *q* = 1 has then to be added to ensure a unit quaternion:

$$\begin{array}{c} \Lambda =q^{T}Bq-\lambda \left(q^{T}q-1\right) \end{array}$$ (11)

Let *s*_*j*_ = *S*_*jj*_. The derivatives of this new formulation can then be written as:

$$\left\{ \begin{array}{ccc} \frac{\partial \Lambda }{\partial q} & = & (B-\lambda I_{4})q\\ \frac{\partial \Lambda }{\partial s_{j}} & = & -q^{T}(\sum_{i}Q_{y_{i}^{\prime }}\frac{\partial P_{\xi_{i}}}{\partial s_{j}})q+s_{j}\sum_{i}{\tilde{x}}_{ji}^{2} \end{array}\right.$$ (12)

Derivative with respect to *q* depends upon *s*_*j*_ and vice versa. Therefore, a direct solution to the problem of minimizing $\tilde{C}\left(q,S\right)$ is difficult to find if not impossible. However, separating the problem between *S* and *q* leads to an alternate optimization scheme, each having an analytical solution.

*Rotation*:

$$\begin{array}{c} \frac{\partial \Lambda }{\partial q}=0\Leftrightarrow \left(B-\lambda I_{4}\right)q=0 \end{array}$$ (13)

Solving this equation amounts finding the eigen vectors of *B*. More precisely, the global minimum $\hat{q}$ is the one associated to the smallest eigen value of *B* as shown in [36], [35].

*Scaling*: (see S1.3)

$$\begin{array}{c} \frac{\partial \Lambda }{\partial s_{j}}=0\Leftrightarrow {\hat{s}}_{j}=\frac{1}{\sum_{i}{\tilde{x}}_{ji}^{2}}q^{T}(\sum_{i}Q_{y_{i}^{\prime }}\frac{\partial P_{\xi_{i}}}{\partial s_{j}})q \end{array}$$ (14)

Now, interestingly the matrices $Q_{y_{i}^{\prime }}\frac{\partial P_{\xi_{i}}}{\partial s_{j}}$ have a quite trivial form. They are all symmetric, only the placing and indexes change (see S1.4). We finally get the following iterative alternate optimization scheme:

- For a fixed value of $\hat{S}$, estimate the new optimal rotation quaternion: $\hat{q}$ as the eigenvector with the smallest eigenvalue of *B*
- For a fixed value of $\hat{q}$, estimate the new optimal scaling matrix $\hat{S}=\mathrm{Diag}\left({\hat{s}}_{1},{\hat{s}}_{2},{\hat{s}}_{3}\right)$ following:
  $${\hat{s}}_{j}=\frac{1}{\sum_{i}{\tilde{x}}_{ji}^{2}}{\hat{q}}^{T}(\sum_{i}Q_{y_{i}^{\prime }}\frac{\partial P_{\xi_{i}}}{\partial s_{j}})\hat{q}$$

## 3 Material and methods

In the previous section, a method to find the optimal anisotropic similarity between two sets of paired points has been depicted. This gives the opportunity to register a database of subjects onto a common reference image using this type of linear transformation to extract scaling factors along chosen orthogonal directions and to study the variation of these factors on different ROIs between populations or among normal subjects.

### 3.1 Material

**Fig 2:**
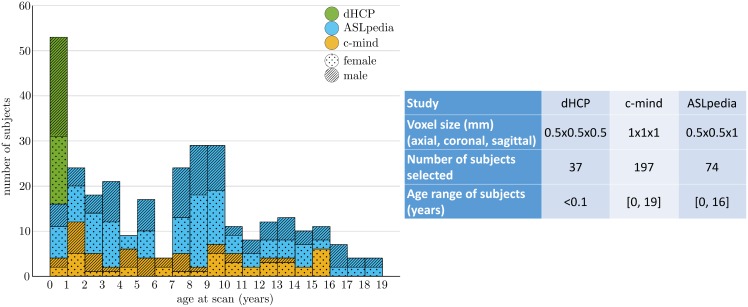
Repartition of the subjects selected from three studies over age.

308 T1-weighted images of healthy subjects between 0 and 19 years old have been used, coming from three different studies: ASLpedia (section 6.1.1), C-MIND (section 6.1.2) and the Developing Human Connectome Project (dHCP) (section 6.1.3). Details on age repartition among databases and on image characteristics are given in Fig 2.

### 3.2 Methods

We developed a pipeline composed of 5 steps to extract scaling factors for 3 orthogonal directions on ROIs from a database of subjects.

1. Choice and construction of the common reference image
2. Segmentation of the common reference image into different ROIs
3. Choice of the constrained directions of scaling for the anisotropic similarity registration
4. Anisotropic similarity registration of a database of subjects to each ROIs of the common reference image to extract relative scaling factors
5. Renormalization of the relative scaling factors to obtain absolute scaling factors

**Fig 3:**
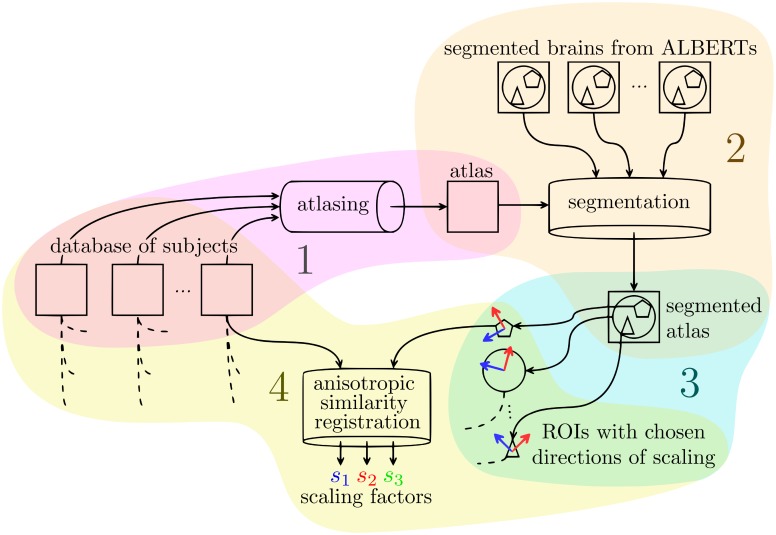
Pipeline for the extraction of scaling factors of a database of subjects using anisotropic similarity registration onto an atlas based on them as common reference image.

The above numbers associated to the steps are also associated to the subsections numbering below and to the numbers in Fig 3.

#### 3.2.1 Creation of the common reference image

For genericity, the common reference image has been chosen to be an atlas made from all the subjects using a modified version of the atlas creation algorithm from [37]. The original method consists in the registration of a dataset of subjects onto a common reference image. Then, the inverse of the average of the local displacements is applied to the average of the registered images to obtain a new reference image. By iterating the process, the atlases (reference images), become less and less biased by the choice of the first reference image in terms of local displacements. However, the unbiasing steps does not take into account affine (global) transformations leading to atlases unbiased up to an affine transformation only. Our method, developed in [38] and available in Anima-Scripts (open source scripts for medical image processing: github.com/Inria-Visages/Anima-Scripts-Public/ - RRID:SCR_017072), takes advantage of the log-Euclidean framework developed in [39] and the Baker-Campbell-Hausdorff formula, mentioned in [40] and [41], allowing to average compositions of affine transformations and diffeomorphisms. This adjustment leads to the creation of atlases up to a rigid transformation. This methods includes in addition mechanisms conceived to improve the robustness of the registration algorithms in case of brains of different sizes through clever initialization and linear transformation decompositions.

#### 3.2.2 Segmentation of the common reference image

**Fig 4:**
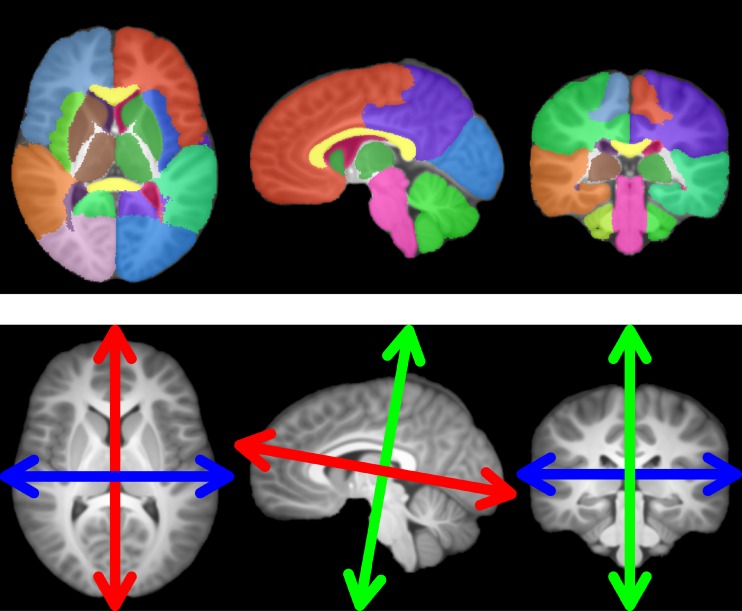
Regions of interest (ROIs) segmented and represented on the common reference image (top), chosen directions of scaling for anisotropic similarity registration defined and represented on the common reference image (bottom).

The atlas has then been segmented into 21 regions of interest (ROIs): whole brain, hemispheres, frontal lobe, parietal lobe, temporal lobe, occipital lobe, basal ganglias, cerebellum, insulas, ventricules, corpus callusum and brainstem. All structures were also separated in their left and right sides. To do this segmentation, ALBERTs manual ones ([42] and [43], see acknowledgments 6.1.4) have been used: 20 brains segmented into 50 regions manually drawn based on MRI brain scans that we fused to obtain the wider desired regions. The T1 weighted images of those brains have been registered onto our atlas through affine then diffeomorphic registration. The outputs have then been used to transfer all the segmentations onto our atlas which have been then merged using majority voting following [44]. The segmented atlas is shown in Fig 4. It should be noted that the 20 brains associated to ALBERTs data were only used for segmentation of the ROIs. They were not used for the creation of the common reference and, not used either as subjects registered with anisotropic similarity.

#### 3.2.3 Choice of the constrained directions of scaling

The fixed scaling directions (characterized by the column vectors of the matrix *U*) are chosen on the reference image such that the first direction (blue in Fig 4) is orthogonal to the mid-sagittal plane (determined using [45]) for symmetry reasons. The other two directions are set using principal component analysis (PCA) on the non zero voxels coordinates projected onto the mid-sagittal plane. The second direction (red in Fig 4) corresponds to the principal direction from the PCA while the third (green in Fig 4) corresponds to the secondary one. three orthogonal directions are now chosen: one through iconic considerations and the other two based on purely geometrical features. In our application, the matrix *U* is the same for all ROIs of the reference image and is defined using the whole brain. However, it is possible to define a different *U* for each ROI independently. Chosen directions of scaling are shown in Fig 4.

These directions have been chosen as a proof of concept. Depending on the purpose of the study, other choice may appear more relevant (see Discussion Section 5).

#### 3.2.4 Anisotropic similarity registration

For each ROI, all subjects undergo an anisotropic similarity registration onto the reference image masked by this ROI. This registration is performed in two steps using in each case our block matching algorithm implemented in Anima (open source software for medical image processing: github.com/Inria-Visages/Anima-Public/ - RRID:SCR_017017):

1. A similarity *T*_*B*_ from whole brain subjects onto whole brain common reference is first estimated.
2. An anisotropic similarity *T*_*C*_ initialized from the previous step output is then computed to bring the subjects onto the atlas masked by the current ROI.

**Fig 5:**
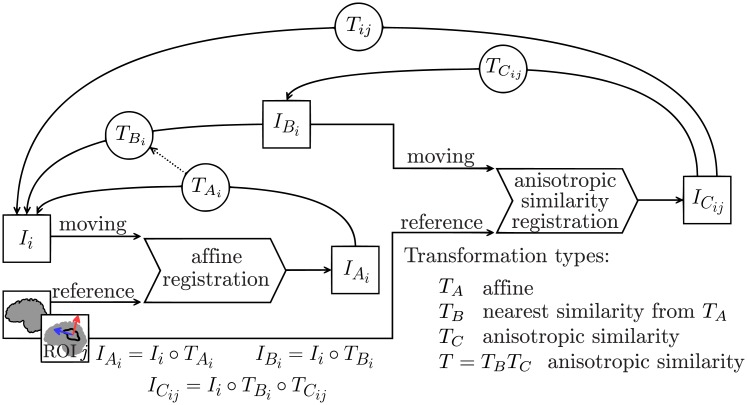
Two steps registration process: First an affine from which a nearest similarity is deduced, then an anisotropic similarity. Transformations are composed and represented with arrows from destination to start since the interpolations occurring in the resampling process are done using the backward mapping. The inverse transformation is actually used on each voxel of the output images to determine the position in the input image from which a value is sampled.

The first transformation, a similarity, is computed indirectly during a process of affine registration. Let *A* be the linear part of an affine transformation *T*_*A*_. We consider the following SVD: *A* = *V DW*^*T*^ with *D* diagonal positive, *V* and *W* unitary matrices. We define *T*_*B*_ (the nearest similarity associated to *T*_*A*_) as the transformation with linear part $B=\bar{d}VW^{T}$ with $\bar{d}$ being the average of the singular values namely the mean of the diagonal of *D*, and translation part $t=\bar{y}-B\bar{x}$. We chose the initialization to be a similarity since the composition of a similarity *T*_*B*_ and an anisotropic similarity *T*_*C*_ associated to a matrix *U* is still an anisotropic similarity associated to the same *U*: *T*_*B*_*T*_*C*_ = (*s*_*B*_*R*_*B*_)(*R*_*C*_*S*_*C*_*U*^*T*^) = (*R*_*B*_*R*_*C*_)(*s*_*B*_*S*_*C*_)*U*^*T*^ = *RSU*^*T*^. The whole registration process is summarized in Fig 5.

#### 3.2.5 Extraction of absolute scaling factors

From the output transformations of the registration step, the relative scaling parameters along the three fixed directions are extracted. Those scaling factors are relative to the reference image. We want to normalize them such that they become about equal to 1 at birth and represent, for other ages, how much a region expanded along the chosen directions since birth. To this end, the fact that all dHCP subjects are very young (less than 1 month) is exploited. The normalization step consist in dividing the relative scaling factors of all the dataset by the average of the ones associated to the dHCP subjects considered as the “root” of the brain expansion. Those new scaling factors will now be considered as absolute scaling factors. At this stage, for each subject, an absolute scaling factor has been determined for each ROI. Those absolute scaling factors are used to model the expansion of the brain toward the chosen directions.

## 4 Experiments and results

### 4.1 Model selection

Several models are traditionally used to represent growth in biostatistics such as the exponential or Weibull models. The second one has been considered by [30] as the best suited to model brain growth in terms of volume. Our case however is different: it can be viewed as a 3-way unidimensional approach. In our quest to find the function best suited to model growth curves for our data, we decided to consider, as a prior, that the brain expansion is stopping at some point. Therefore, we restricted the spectrum to functions that have a horizontal asymptote at infinity. The selected candidates to model brain growth in the chosen directions are the following:

- Rational with polynomials of degree 1 as numerator and denominator: $y=\frac{ax+b}{x+c}$
- Weibull: $y=a-be^{-cx^{d}}$
- Gompertz: $y=ae^{-be^{-cx}}$
- Exponential: *y* = *a* + *be*^−*cx*^

For each candidate, the optimal coefficients are estimated through nonlinear regression using the Levenberg-Marquardt iterative weighted least squares algorithm from [46]. In this process, weights are chosen to compensate for local gender repartition. For each subject *i*, a window of width *l* = 2 years centered on the subject age is considered. Let *n*_*f*_, *n*_*m*_ and *n* be the number of female, male and total subjects respectively in that window. A correction coefficient $c_{f}=\frac{n_{m}}{n}$ is applied if *i* is a female and $c_{m}=\frac{n_{f}}{n}$ if *i* is a male. Let {*y*_1_, …, *y*_*n*_} be the observations (i.e. here the obtained scaling factors), $\bar{y}$ be the average of those and $\left\{{\hat{y}}_{1},\ldots ,{\hat{y}}_{n}\right\}$ be the fitted values.

Based on these statistics, the chosen candidate for the modeling will be the one that best satisfies a criterion quantifying the goodness of fit. This indicator should evaluate the accuracy of the model i.e. how close the model is to the observation while discouraging overfitting. It therefore consists in a tradeoff between accuracy and parsimony. It has been shown in [47] that the coefficient of determination is not, at least when considered alone, an appropriate measure for the goodness of fit in the case of nonlinear model selection. A more adapted goodness of fit for nonlinear model selection is the Akaike information criterion (AIC) developed in [48] and [49]. Based on information theory, it proposes to estimate the information loss induced by each candidate model to represent an unknown process that supposedly generated the data as shown in [50]. This is made possible through the estimation of the Kullback-Leibler divergence related to the maximized log-likelihood. AIC is defined by: $AIC=-2p-2\ln(\hat{L})$, where $\hat{L}$ is the maximum likelihood of the candidate model and the first term penalizes a large number of parameters *p*. Therefore, the preferred model among the candidates is the one with the lower AIC. Note that AIC of a model taken alone is meaningless, it makes sense only when compared to the one of the other models. This is why it is recommended to consider it along with another statistic that quantifies the error between the model and the data like mean of squared errors (MSE): $MSE=\frac{1}{n}\sum_{i}{\left(y_{i}-{\hat{y}}_{i}\right)}^{2}$ which is the average of the residuals. A corrected version of the AIC has been developed to avoid overfitting in the case of small sample sizes: $AIC_{c}=AIC+\frac{2p(2p+1)}{n-p-1}$. To facilitate the interpretation that can be quite obscure using raw AIC, following [51], it is possible to transform those values into conditional probabilities for each model called Akaike weights. Defined for each model *i* by $w_{i,AIC}=\frac{e^{-\frac{1}{2}\left(AIC_{i}-AIC_{min}\right)}}{\sum_{j}e^{-\frac{1}{2}\left(AIC_{j}-AIC_{min}\right)}}$, those weights represent the probability for each candidate *i* to be the best suited in the sense of AIC to model the data among all the candidates.

**Fig 6:**
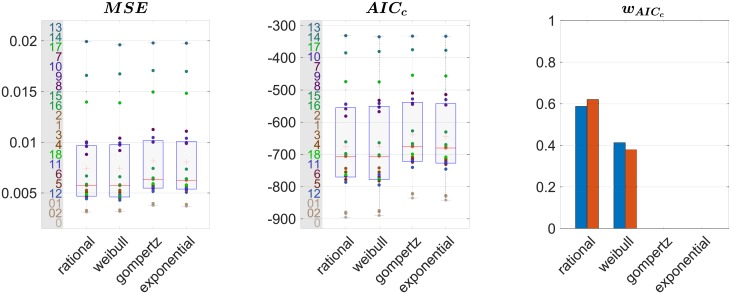
Goodness of fits for each candidate to model the ouputed scaling factors averaged in the 3 directions. Boxplots are performed along the ROIs, ROI IDs are displayed on the left. Akaike weights are computed on mean (blue) and median (red) AIC_c_.

All the goodness of fit depicted above as well as MSE have been evaluated for each of our candidates to model the scaling factors for each ROI. We present the results of this evaluation in Fig 6. The Gompertz and exponential models are largely below the other two. Even though the Weibull model behaves relatively well, the rational one shows better scores whatever the tested goodness of fit.

### 4.2 Directional growth curves

**Fig 7:**
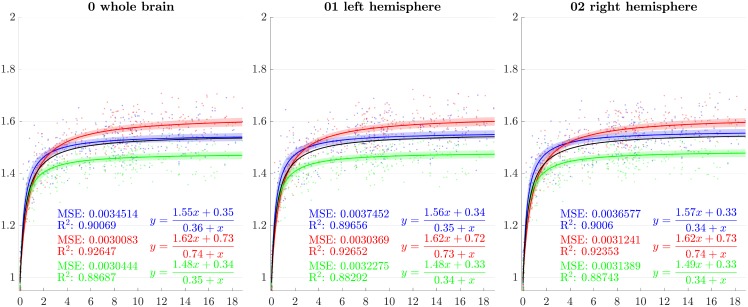
Resulting scaling factors as a function of the age in years for differents ROIs, along direction 1 (blue), 2 (red), 3 (green). Fitted using rational model together with 99% confidence intervals for fitted values, Black curves represent the average models along the three directions.

**Fig 8:**
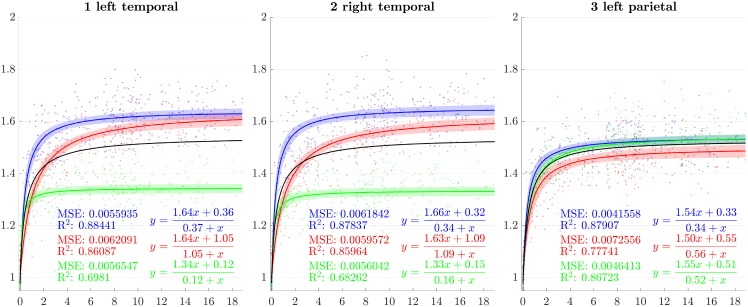
Resulting scaling factors as a function of the age in years for differents ROIs, along direction 1 (blue), 2 (red), 3 (green). Fitted using rational model together with 99% confidence intervals for fitted values, Black curves represent the average models along the three directions.

**Fig 9:**
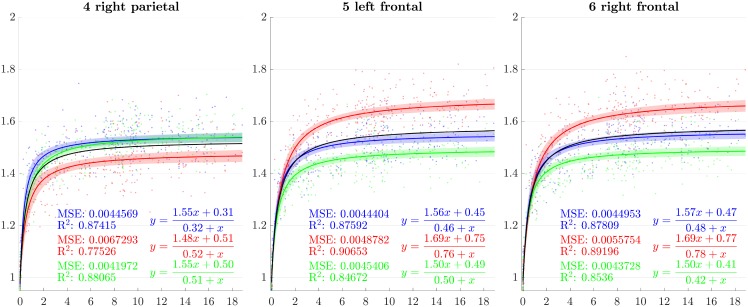
Resulting scaling factors as a function of the age in years for differents ROIs, along direction 1 (blue), 2 (red), 3 (green). Fitted using rational model together with 99% confidence intervals for fitted values, Black curves represent the average models along the three directions.

**Fig 10:**
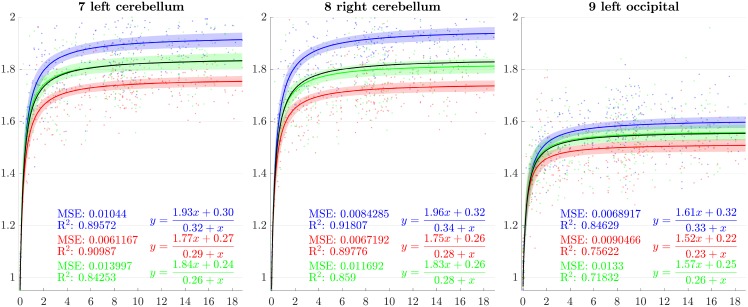
Resulting scaling factors as a function of the age in years for differents ROIs, along direction 1 (blue), 2 (red), 3 (green). Fitted using rational model together with 99% confidence intervals for fitted values, Black curves represent the average models along the three directions.

**Fig 11:**
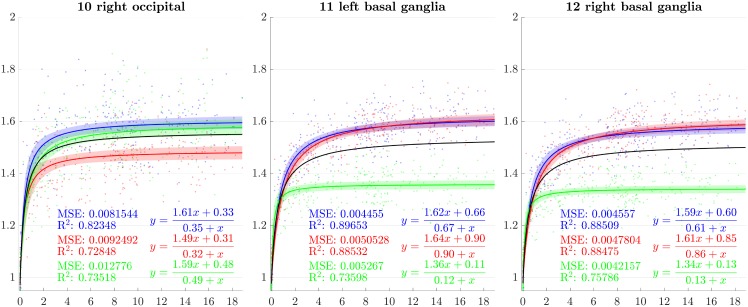
Resulting scaling factors as a function of the age in years for differents ROIs, along direction 1 (blue), 2 (red), 3 (green). Fitted using rational model together with 99% confidence intervals for fitted values, Black curves represent the average models along the three directions.

**Fig 12:**
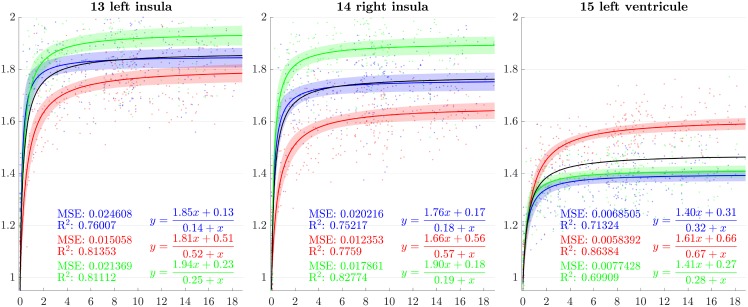
Resulting scaling factors as a function of the age in years for differents ROIs, along direction 1 (blue), 2 (red), 3 (green). Fitted using rational model together with 99% confidence intervals for fitted values, Black curves represent the average models along the three directions.

**Fig 13:**
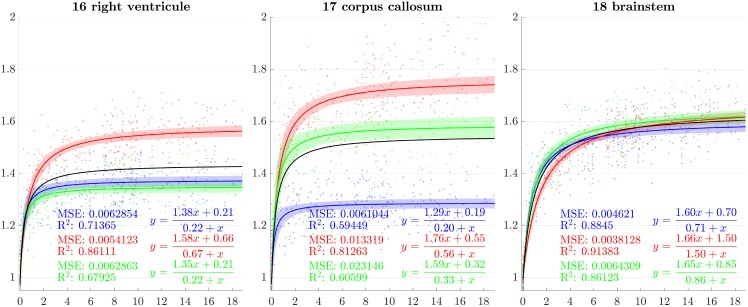
Resulting scaling factors as a function of the age in years for differents ROIs, along direction 1 (blue), 2 (red), 3 (green). Fitted using rational model together with 99% confidence intervals for fitted values, Black curves represent the average models along the three directions.

From the previous sections, scaling factors in each direction for each ROI are now modeled using a rational function with polynomials of degree 1 as numerator and denominator chosen after model selection. Results for all regions studied are presented in Figs 7–13. The method presented by [52] is used to compute simultaneous 99% confidence intervals for fitted values. The black curve represents the average brain growth computed as the mean of the directional models (Figs 7–13).

### 4.3 Male vs female comparison

Gender, like age, is a characteristic of the subjects available in all the studies we considered. The aim of this section is to evaluate if differences in terms of scaling factor can be found between genders. We divided our data into four age-intervals based on the age of the subjects. The first one contains dHCP participants (newborns), the second one is composed of all non-newborn subjects between 0 and 6 years old, the third one between 6 and 12 and the fourth older to 12. Repartition of the subjects in terms of gender, age class and study is shown in Table 1.

**Table 1:** Repartition of the subjects in term of age class, gender and study.

		dHCP		(0, 6]		(6, 12]		>12		
male	dHCP	22	22	51	0	48	0	26	0	147
	c-mind		0		29		37		24	
	ASLpedia		0		22		11		2	
female	dHCP	15	15	54	0	57	0	35	0	161
	c-mind		0		43		43		21	
	ASLpedia		0		11		14		14	
		37		105		105		61		308

**Fig 14:**
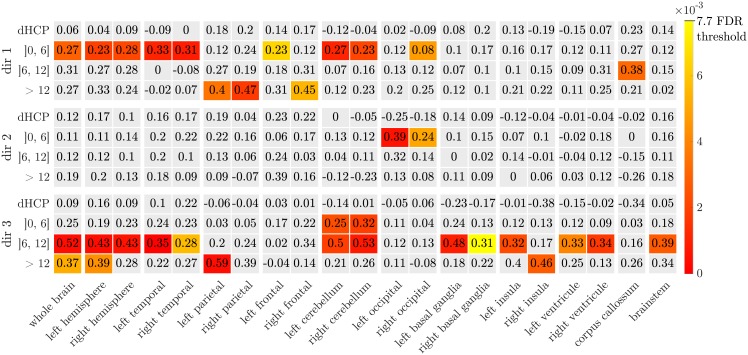
Male vs female comparison using Wilcoxon-Man-Whitney U-test and *H*_0_: The distribution of the scaling factors of males and females are equal, *H*_1_: The distributions of males and females are different. In color: p-values for *H*_0_ rejection for FDR at level 5% (Benjamini and Hochberg method). Numerically: the size of the effect *d* for each test.

For each of these age-intervals, and each of the chosen scaling directions, and each ROI, we performed a test to evaluate if the scaling factors for male subjects are greater than scaling factors for female subjects. Since these data are not normally distributed in those subdivisions, we used two-tailed Wilcoxon-Man-Whitney U-tests. For each of those tests, the null hypothesis *H*_0_ is the following: the distribution of the scaling factors between males and females are equal, while the alternative hypothesis *H*_1_ states: the distributions of males and females are different. We performed 252 tests in total: 4 age-intervals × 21 ROIs × 3 directions whose results are shown in Fig 14.

A type 1 error, or false positive, occurs when *H*_0_ is incorrectly rejected. Since we are doing multiple comparisons, rejecting *H*_0_ based on the risk of type 1 error *α* = 5%, may lead in our case to an expected number of false positives greater than 12. Instead of using *α*, we therefore adopted the false discovery rate (FDR) that controls the proportion of false positives among the tests where *H*_0_ has been rejected. Therefore, we stated the acceptance or rejection of *H*_0_ based on a FDR at level 5%. This has been done using Benjamini and Hochberg procedure from [53] and corresponds to reject *H*_0_ when the p-value is less than 0.0077 (Fig 14). FDR has been preferred to family-wise error rate (FWER), that controls the risk of at least 1 false positive among the whole family of tests, because of the over-conservatism of this last type of procedure leading to poor test power (probability of correctly rejecting *H*_0_). Additionally, we calculated, for each test, the effect size *d* following: $d=\frac{median\left(\left\{S_{m}\right\}\right)-median\left(\left\{S_{f}\right\}\right)}{\sigma \left(\left\{S_{m}\right\}\right)+\sigma \left(\left\{S_{f}\right\}\right)}$ (Fig 14), where {*S*_*m*_} (resp. {*S*_*f*_}) is the set of scaling factor of males (resp. females) used for the test. We preferred the use of median instead of mean due to the fact that we do not know the distribution of the data a priori and we performed ranksum type tests.

For all the tests that lead to a rejection of the null hypothesis, scaling factors were higher for males both in terms of means and medians. Tests show that scaling factors of males seem higher in the second age class (0-6), brainwise and mainly in temporal and cerebellum areas along the direction 1. This is also notable in the same regions between 6 and 12 years, this time along direction 3. For the older class (12-19), this phenomenon essentially appears brainwise along the direction 3 and in the parietal lobes along direction 1.

### 4.4 Influence of the common reference

**Fig 15:**
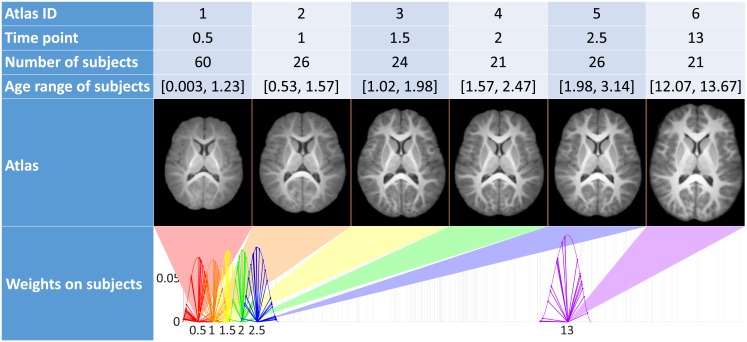
Characteristics of the 6 atlases used as reference image (time is displayed in years).

To evaluate the influence of the common reference image, the whole process described previously is reproduced using six different reference images. Those are atlases for six arbitrary chosen time-points *t*_1_, …, *t*_6_ using subjects among the population depicted in Section 3.1. Time-points are chosen such that five of them cover the period in which the majority of the brain expansion occurs, the last is positioned later, in a stabilized area. Following [38], an atlas for a time-point *t*_*i*_ is created using only the subjects with ages in a range around *t*_*i*_ (in Fig 15, line 4). The contributions of those subjects are then weighted using a weight function (plotted in Fig 15, line 6) such that subjects closer in age to *t*_*i*_ are given more importance. The six outputed atlases (presented Fig 15) are different in terms of shape and intensity, they will be used to challenge the robustness of our method to extract scaling factors.

The method developed in section 3.2 is used for each of these reference images, on which directions of scaling for the anisotropic similarity registration have been established the same way. A scaling factor *s*(*i*, *j*, *d*, *k*) is thus computed for each ROI *j* of each subject *i* based on each reference image *k* along each chosen direction *d*. To quantify the influence of the reference image on absolute scaling factors, the results, using the six reference images previously depicted, are compared through two approaches:

1. A pairwise study to evaluate whether or not reference atlases closer to each other in age are more likely to generate closer results.
2. A study of the standard deviation among results for all reference atlases to evaluate how far they are from the average results.

#### 4.4.1 Study of pairwise distances between scaling factors by reference images in each direction for each ROI

Our aim is to determine whether or not reference images closer to each other (atlases at shorter temporal distances) are more likely to generate less important absolute differences between their results. We therefore compute the absolute difference of the resulting scaling factors between each pairwise combinations of reference images. Then, those distances are normalized by the average of corresponding scaling factors between the two atlases such that it can be seen as a percentage of it (relative distance). The relative distance between scaling factors from reference atlases *k* and *l* is then computed as:

$$\begin{array}{c} D_{k,l}\left(i,j,d\right)=2\frac{\left|s(i,j,d,k)-s(i,j,d,l)\right|}{s(i,j,d,k)+s(i,j,d,l)} \end{array}$$ (15)

**Fig 16:**
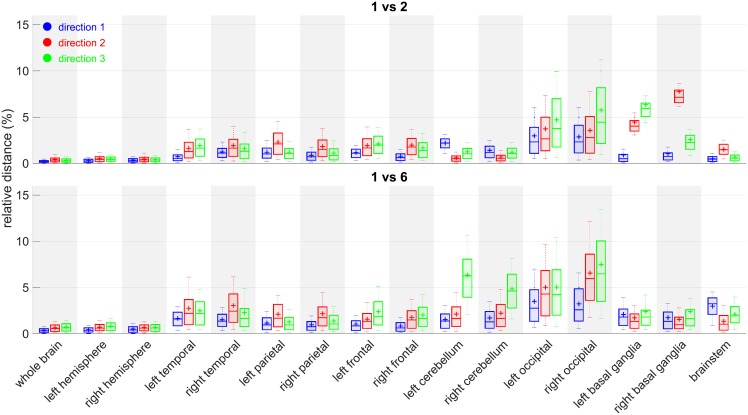
Relative distances between reference atlas 1 and 2 (top), 1 and 6 (bottom). Boxplots among subjects for each ROI *j*, each direction *d*: *boxplot*(*D*_*k*,*l*_(., *j*, *d*)).

After examination of all the pairwise combinations, the temporal distance between the reference images does not seem to have an impact on the distance of the scaling ratios associated to each other (Fig 16). The highest median of relative distance happens to be between atlases 2 and 5 for right basal ganglia, but does go above 8% of difference.

#### 4.4.2 Study of the standard deviation among reference images in each direction for each ROI

This method gives an average measure of the distances between the results for each atlas and the average results. Those distances are normalized by the average of corresponding scaling factors of all the atlases. The relative standard deviation between scaling from all reference atlases is then computed as:

$$\begin{array}{c} D\left(i,j,d\right)=\frac{\sigma (s(i,j,d,.))}{\bar{s(i,j,d,.)}} \end{array}$$ (16)

**Fig 17:**
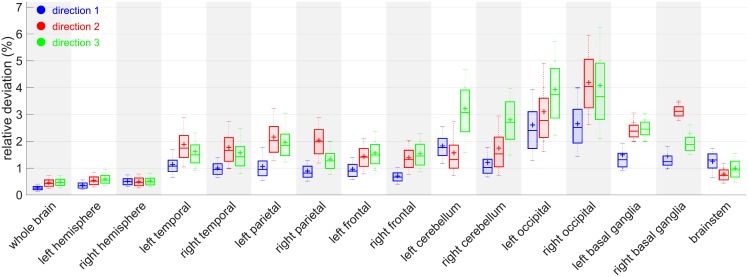
Relative standard deviation between reference atlases. Boxplots among subjects for each ROI *j*, each direction *d*: *boxplot*(*D*(., *j*, *d*)).

The graphs (Fig 17) suggest that the method, when applied to large regions such as whole brain and hemispheres, is really robust to reference image change. Occipital lobes and cerebellum however seem to be more vulnerable areas. Those two regions share a common border and we believe that the segmentation process is a crucial step in that case. The cerebellum position indeed varies quickly in early stages of life and our decision to use segmentations based on neonates can be a bit inadequate for this area in particular. We also think that the way we chose to define the constrained directions of scaling (especially those using purely geometrical considerations through PCA on voxel coordinates) may not be the best suited for robustness in those areas. More anatomical features could lead to even smaller influence of the reference image.

## 5 Discussion

### 5.1 Comparison of the results to the literature

Our method aims at characterizing directional growth at the scale of a ROI through the extraction of scaling factors, which are easily interpretable measures. In most longitudinal analyses of brain development, the focus is on volumetry. This type of measurement reflects the global growth of a region but lacks characterization of directional expansion. Other approaches are based on cortical surface curvature as in [12], [54] or [55] to reflect gyri and sulci widening or deepening, giving information about directional expansion at a more local scale.

Values from volumetric measurements cannot be directly compared to our scaling factors: they are three-times unidimensional whereas volume is not. Our anisotropic scaling factors should instead be seen as a complementary information to the volume data. However, there are still some aspects that we can compare:

- The pace of growth: is the growth monotonous or does it increase or decrease in speed at given ages? Are there regions that seem to evolve at a quicker pace than the others do?
- The asymptote: at what age does the growth reach a peak and begins to stagnate? Are there regions that seem to reach a higher growth asymptote than others do?

Since the anisotropy is a missing information in volumetric studies, it is appropriate to use the average models of the three directions (black curves in Fig 7) for the comparisons. In [13], [30], [23] and [18], the pace of the whole brain volume curves is quite similar to our findings, showing a strong initial increase followed by a decline around 2 years. In [25], it is also noticeable that, analogously to our results, the cerebellum grows by a much more important factor than the brain as a whole. In [18], basal ganglia grow by a factor comparable to the whole brain which is also consistent with our results.

### 5.2 Implementation considerations

#### 5.2.1 Directions of scaling

The choice of the scaling directions, characterized by the matrix *U*, is crucial. These three directions can be different for each ROI, they are defined on the reference image and must be orthogonal. The brain being a rather symmetrical organ about its mid-sagittal plane, choosing a direction orthogonal to it seems natural. Given that, there is no obvious choice for the other two. Using PCA on non-zeros voxel coordinates of the reference image (atlas of the population in our case) projected on mid-sagittal plane (since the last two directions have to be orthogonal to the first one) seemed to be quite intuitive. This corresponds to the principal sagittal directions of the average model of the population. However, it is based on purely geometric features ignoring iconic or anatomical considerations. This choice must above all be seen as a proof of concept and should not be considered as the one to be necessarily adopted. A future work could be to define, for a given purpose, a criterion of goodness for the chosen directions and, to optimize to find the directions that best satisfy this criterion. A more anatomically-oriented approach could be to ask a medical expert to point, on the reference image, the anterior commissure—posterior commissure (AC-PC) line. This has been well adopted as a standard by the clinical neuroimaging community even though it is mostly a convention for visualization and at the cost of introducing a human interaction or a preprocessing step. This method is therefore very flexible in the choice of the scaling directions and the ROIs, yet it has shown oneself robust concerning the choice of the common reference image.

#### 5.2.2 ROI segmentation

Although it does not call into question the method itself, there is room for improvements in the way we segmented the ROIs. The main difficulty is to find a method that is reproducible while being adaptable to brains from subjects scanned across a wide range of ages, which induces a large variability in contrast and shape. It has been shown in [56] that the automatic segmentation of 1 year old subjects was more successful when using prior segmentations of 2 years old than when using prior segmentations of adults. In addition, [57] showed that, in the context of multi-atlas segmentation, similarity selection of the atlases increases significantly the accuracy compared to the fusion of random sets of atlases. In the same paper, it has also been established that using an age-based selection gives similar results to similarity selection. Those results support the assertion that segmentation accuracy is improved by using age-adapted atlases. However, the concern of producing segmentations on a longitudinal database that are adapted to the maturation of each brain while being consistent among the subjects is still, to our knowledge, an open question. Our choice to use ALBERTs atlases to segment all the subjects regardless of their age was driven by a desire of consistency/reproducibility to the expense of age-adaptation since we did not find methods offering a satisfying trade-off between the two.

#### 5.2.3 Normalization step

We have used a normalization step instead of the raw scaling factors relative to the reference image. Even though it is in a way more abstract, the relative scaling factors can actually be used as such. A subject with abnormalities that would show itself atypical in terms of scaling factors compared to the normal model would be so, with or without normalization. Now it is indeed more tangible for interpretation to have a concrete base like birth. In that case, scaling factors have an intuitive meaning since they quantify how much a region grows along a given direction since birth. In the event that there is no neonatal data to normalize the relative results, the following could be done:

- If a model can be fitted from the relative scaling factors, it might be possible to extrapolate values for birth and therefore use them for normalization.
- If not, relative scaling factors may be used or another base should be considered. Taking values in the growth stabilization zone may also be considered as it gives easily interpretable results as well.

That being said, neonatal MRI data are nowadays quite abundant in open access, especially structural images. The dHCP team (see Acknowledgment 6.1.3) announced in September their second release with over 500 neonates. Our normalization can therefore be performed when using this data.

#### 5.2.4 Image quality

Images used in the experiments are of good quality compared to what can be commonly found in clinical routine. Registration is sensitive to image quality but it is mostly the case in the context of non-linear registration where there are many degrees of freedom and very local displacements are involved. Even if the image quality can have an impact, the sparsity in terms of degrees of freedom and the global aspect of the anisotropic registration make it much more resilient about image size and quality. In addition, the bigger is the size of the ROI is, the less sensitive to image quality the result will be. The registration algorithm being based on block-matching, the ROI must be chosen such that their sizes are at least one order larger than the size of the blocks. A block being a neighborhood of voxels, the poorer the image quality, the larger the ROI must be and too narrow structures should be avoided. Finally, conducting the study at a group scale attenuates the influence of potential registrations errors induced by a poor image quality.

### 5.3 Clinical applications

We focused on the expansion of structures of a database of healthy subjects but we can also imagine using this method for patients. Intra-individual surveys are also possible, for subjects that had multiple scans through time, to monitor the evolution of a brain sub-region or any part of the body and infer the way it is going to expand.

Brain morphometric measurements have been performed on infants with deformational plagiocephaly and controls in [58]. They found that volume analysis does not allow the distinction between cases and controls. However, their unidimensional measurements based especially on distances between landmarks were more successful. Those nevertheless necessitate point picking on all subjects and may be sensitive to human error. Considering this, it could be interesting to try, in future work, our automatic method (only the computation of directions for each ROI on the common reference is needed) on this kind of pathology. Some chosen directions could for example be colinear to the line segments that have proved to be relevant in this paper. Those measurements might be linked to developmental delays that affect infants with deformational plagiocephaly.

## Conclusion

We have presented a method that allows the extraction of regional and global scaling factors along arbitrary chosen orthogonal directions. This is done through linear registration using a 9 dof transformation, anisotropic similarity, which is an affine transformation with constrained directions of scaling.

The main methodological contribution of this paper concerns the resolution of the problem of finding the optimal anisotropic similarity that best matches two sets of paired points. This result has made possible the development of a block-matching registration algorithm based on this transformation.

Given this new type of registration, our second contribution was to map a database of subjects between 0 and 19 years old using anisotropic similarity onto a common reference image on which the constrained directions of scaling of our choosing have been fixed. Registrations have been performed brainwise and ROI wise (lobes, cerebellum, basal ganglias…). For genericity purpose, we chose this reference image to be an atlas made from the subjects. Based on symmetry and geometrical considerations, we defined the same constrained directions of scaling for all ROIs even though it is possible to choose different ones for each. As an output, we obtained for each subject, for each ROI, for each chosen direction a scaling, a scaling factor that we normalized such that it represents an expansion factor from birth.

Those scaling factors have been used to model the anisotropic development of the brain. After model selection, it has been determined that rational function with polynomials of degree 1 as numerator and denominator is the best suited among the tested candidates for that modeling. Curves representing scaling factors as a function of the age for each ROIs, each chosen direction, along with associated confidence intervals have then beeen computed on a combination of four databases.

Tests to determine the influence of gender in those scaling factors have been performed for different age-intervals. Finally, two experiments have been conducted to evaluate the influence of the aforementioned common reference image. The results have shown small relative differences depending on the choice of the reference image leading to the conclusion that the method is robust in that aspect.

## 6 Acknowledgments

### 6.1 Databases

#### 6.1.1 ASLpedia

A retrospective ASL study on routine pediatric MRI performed at Rennes University Hospital Neuropediatric radiology Department.

#### 6.1.2 C-MIND

Data Repository created by the C-MIND study of Normal Brain Development. This is a multisite, longitudinal study of typically developing children from ages newborn through young adulthood conducted by Cincinnati Children’s Hospital Medical Center and UCLA and supported by the National Institute of Child Health and Human Development (Contract HHSN275200900018C). A listing of the participating sites and a complete listing of the study investigators can be found at: https://research.cchmc.org/c-mind.

#### 6.1.3 The Developing Human Connectome Project (dHCP)

Led by King’s College London, Imperial College London and Oxford University, aims to make major scientific progress by creating the first 4-dimensional connectome of early life.

https://data.developingconnectome.org/.

#### 6.1.4 ALBERTs

See [42] and [43] for details about segmentations. Copyright Imperial College of Science, Technology and Medicine and Ioannis S. Gousias 2013. All rights reserved. https://brain-development.org/brain-atlases/neonatal-brain-atlases/neonatal-brain-atlas-gousias/.

## Supporting information

S1 Appendix Detailed calculations for optimizing anisotropic similarity between two sets of paired points. (PDF)
