# Supplementary material for: Regional brain development analysis through registration using anisotropic similarity, a constrained affine transformation
Source: PLoS One. 2020 Feb 24;15(2):e0214174. doi: 10.1371/journal.pone.0214174 (PMC7039415; doi:10.1371/journal.pone.0214174)
Supplement: S1 Appendix — (PDF) [file pone.0214174.s001.pdf]

S1 Appendix - Detailed calculations for optimizing anisotropic similarity between two sets of paired points

S1.1

Let R be a rotation matrix. Then, an associated unit quaternion q is defined such as if $Rx = q * x * \bar{q}$. Thus:

$$\begin{aligned}\tilde{C}(q,S) &= \sum_i \|y_i' - q * \xi_i * \bar{q}\|^2 \\ &= \sum_i \|y_i' * q - q * \xi_i\|^2\end{aligned} \tag{17}$$

S1.2

If p is a vector, the associated quaternion is pure: $p_1 = 0$ which implies that Q_p and P_p are skew-symmetric. Yet y_i' and ξ_i are vectors, thus:

$$\begin{aligned}\tilde{C}(q,S) &= \sum_i \|y_i' * q - q * \xi_i\|^2 \\ &= q^T \left(\sum_i \left(Q_{y_i'} + P_{\xi_i}\right)^T \left(Q_{y_i'} + P_{\xi_i}\right) \right) q \\ &= q^T \left(-\sum_i \left(Q_{y_i'} + P_{\xi_i}\right)^2 \right) q\end{aligned} \tag{18}$$

S1.3

If p is a vector, the associated quaternion is pure: $p_1 = 0$ which implies that Q_p and P_p are skew-symmetric and $Q_p^2 = P_p^2 = -p^T p I_4$.

Yet y_i' and ξ_i are vectors, thus:

$$\begin{aligned}\tilde{C}(q,S) &= -q^T \left(\sum_i \left(Q_{y_i'} + P_{\xi_i}\right)^2 \right) q \\ &= -q^T \left(\sum_i \left(Q_{y_i'}^2 + 2Q_{y_i'} P_{\xi_i} + P_{\xi_i}^2\right) \right) q \\ &= -q^T \left(\sum_i \left(-{y_i'}^T y_i' I_4 + 2Q_{y_i'} P_{\xi_i} - \xi_i^T \xi_i I_4\right) \right) q \\ &= -q^T \left(\sum_i \left(-{y_i'}^T y_i' I_4 + 2Q_{y_i'} P_{\xi_i} - \tilde{x}_i^T S^2 \tilde{x}_i I_4\right) \right) q\end{aligned} \tag{19}$$

Thus:

$$\frac{\partial \tilde{C}}{\partial s_j} = -q^T \left(\sum_i (2Q_{y'_i} \frac{\partial P_{\xi_i}}{\partial s_j} - 2\tilde{x}_i^T s_j E_{jj} \tilde{x}_i) \right) q$$

E_{jj} being the matrix with a 1 at the intersection of the jth row and the jth column and 0 elsewhere.

$$= -2q^T \left(\sum_i Q_{y'_i} \frac{\partial P_{\xi_i}}{\partial s_j} \right) q + 2q^T \left(s_j \sum_i \tilde{x}_{ji}^2 \right) q$$

yet $\sum_i \tilde{x}_{ji}^2$ scalar and $q^T q = 1$

$$= -q^T \left(\sum_i Q_{y'_i} \frac{\partial P_{\xi_i}}{\partial s_j} \right) q + s_j \sum_i \tilde{x}_{ji}^2$$

$$\frac{\partial \tilde{C}}{\partial s_j} = 0 \Leftrightarrow \hat{s}_j = \frac{1}{\sum_i \tilde{x}_{ji}^2} q^T \left(\sum_i Q_{y'_i} \frac{\partial P_{\xi_i}}{\partial s_j} \right) q \tag{20}$$

S1.4

$$Q_{y'_i} \frac{\partial P_{\xi_i}}{\partial s_1} = \tilde{x}_{1i} \begin{pmatrix} y'_{1i} & 0 & -y'_{3i} & y'_{2i} \\ 0 & y'_{1i} & y'_{2i} & y'_{3i} \\ -y'_{3i} & y'_{2i} & -y'_{1i} & 0 \\ y'_{2i} & y'_{3i} & 0 & -y'_{1i} \end{pmatrix}, \quad Q_{y'_i} \frac{\partial P_{\xi_i}}{\partial s_2} = \tilde{x}_{2i} \begin{pmatrix} y'_{2i} & y'_{3i} & 0 & -y'_{1i} \\ y'_{3i} & -y'_{2i} & y'_{1i} & 0 \\ 0 & y'_{1i} & y'_{2i} & y'_{3i} \\ -y'_{1i} & 0 & y'_{3i} & -y'_{2i} \end{pmatrix}$$
$$\text{and} \quad Q_{y'_i} \frac{\partial P_{\xi_i}}{\partial s_3} = \tilde{x}_{3i} \begin{pmatrix} y'_{3i} & -y'_{2i} & y'_{1i} & 0 \\ -y'_{2i} & -y'_{3i} & 0 & y'_{1i} \\ y'_{1i} & 0 & -y'_{3i} & y'_{2i} \\ 0 & y'_{1i} & y'_{2i} & y'_{3i} \end{pmatrix} \tag{21}$$
